# The Impact of Intravenous Versus Intra-Arterial Heparin Administration on Radial Artery Spasm During Transradial Coronary Angiography

**DOI:** 10.3390/diagnostics16111656

**Published:** 2026-05-28

**Authors:** Eyyup Tusun, Mehmet Han Mercan, Müslüm Karakaş, Necmettin Korucuk, Veysel Tosun

**Affiliations:** 1 Department of Cardiology, Mehmet Akif İnan Training and Research Hospital, Şanlıurfa 63200, Turkey; mehmethanm@yahoo.com (M.H.M.); drmuslumkarakas@gmail.com (M.K.); veyseltosun8810@gmail.com (V.T.); 2 Department of Cardiology, Antalya City Hospital, Antalya 07080, Turkey; necmettinmd@gmail.com

**Keywords:** transradial access, radial artery spasm, heparin, coronary angiography, complication

## Abstract

**Background/Objectives:** Radial artery spasm (RAS) is an important complication during transradial coronary angiography that may negatively affect procedural success and reduce patient comfort. The aim of this study was to comparatively evaluate the effects of intravenous (IV) and intra-arterial (IA) heparin administration on the development of RAS. **Methods:** This prospective, observational parallel-group cohort study included a total of 223 patients undergoing transradial coronary angiography. Patients were divided into two groups, receiving either IV heparin (*n* = 77) or IA heparin (*n* = 146). All patients received a standard dose of unfractionated heparin (5000 IU) and an IA spasmolytic cocktail consisting of 2.5 mg verapamil and 100 mcg nitroglycerin. RAS was defined as pain during the procedure, resistance during catheter manipulation, or the need for crossover. Logistic regression analysis and receiver operating characteristic (ROC) curve analyses were performed. **Results:** RAS developed in 40 of 223 patients (17.9%). The incidence of RAS was significantly higher in the IA heparin group than in the IV heparin group (23.3% [34/146] vs. 7.8% [6/77]; *p* = 0.004). Crossover to femoral access due to severe spasm was observed only in the IA group (6.2% [9/146] vs. 0% [0/77]; *p* = 0.026). Patients who developed RAS were younger, required a greater number of catheters, had longer angiography duration, and were exposed to a higher total radiation dose (*p* < 0.05 for all). In ROC analysis, the number of catheters used and angiography duration showed comparable performance in predicting RAS. In multivariable logistic regression analysis, IA heparin administration and the number of catheters used were identified as independent predictors of RAS. **Conclusions:** During transradial coronary angiography, intravenous heparin administration is associated with a significantly lower frequency of RAS and a reduced need for femoral crossover compared with intra-arterial administration. IV heparin may represent an easily applicable strategy for RAS prevention, although causality cannot be established from this observational study.

## 1. Introduction

The transradial approach in coronary angiography and percutaneous coronary interventions (PCIs) was first described by Campeau in 1989 and has progressively replaced the transfemoral approach in interventional cardiology since its subsequent use by Kiemeneij for coronary stenting in the early 1990s [1,2]. Compared with transfemoral access, the transradial route has been shown to significantly reduce the risk of major bleeding and vascular complications [3]. In addition, the transradial approach allows earlier patient mobilization and generally improves patient comfort. Large multicenter randomized controlled trials have demonstrated that transradial access is at least as safe as femoral access in patients with acute coronary syndromes and may reduce net adverse clinical events and even mortality [4,5]. In light of this strong evidence, guidelines from the European Society of Cardiology (ESC) and the American Heart Association (AHA) recommend transradial access as the preferred vascular access route in both ST-segment elevation myocardial infarction (STEMI) and non-ST-segment elevation acute coronary syndrome (NSTE-ACS), provided that the procedure is performed by an experienced operator [6,7,8].

Despite its advantages, the transradial approach has unique technical challenges and complications. Among these, radial artery spasm (RAS) is one of the most important because it directly affects procedural success and may be highly painful for the patient. RAS is related to the rich distribution of alpha-1 adrenergic receptors in the radial artery, which renders the vessel wall more sensitive to circulating catecholamines [9]. Friction caused by catheter advancement within the vessel, multiple puncture attempts, or patient anxiety during the procedure may trigger endothelial dysfunction, leading to sudden and marked vasoconstriction, namely spasm [10,11]. In the literature, the reported incidence of RAS ranges from 4% to 20%, depending on the criteria used for its definition and the characteristics of the patient population [12]. When severe spasm develops, catheter advancement may become impossible, procedural duration may be prolonged, the patient’s radiation exposure may increase, and, ultimately, the procedure may fail, necessitating crossover to femoral access.

Several pharmacological strategies have been developed to prevent RAS. Intra-arterial administration of calcium channel blockers, particularly verapamil or diltiazem, and nitrates such as nitroglycerin, either alone or in combination as a “spasmolytic cocktail,” has become standard practice in many catheterization laboratories [13,14]. In addition, heparin is routinely administered during transradial procedures; however, the potential effect of its route of administration on the development of spasm has not been adequately clarified in the literature. Heparin may be administered intra-arterially immediately after sheath insertion or systemically through the intravenous route. Although both routes have previously been shown to be similarly effective in preventing radial artery occlusion (RAO), it remains unclear whether IA heparin may increase the risk of RAS through local chemical irritation of the vessel wall [15].

The aim of our study was therefore to comparatively investigate the effects of intravenous and intra-arterial heparin administration on the frequency of RAS in patients undergoing transradial coronary angiography. We hypothesized that IV heparin administration, by avoiding local vessel wall irritation, would be associated with a lower incidence of RAS than IA administration.

## 2. Materials and Methods

This study was designed as a prospective, single-center, observational, parallel-group cohort study. The study protocol was conducted in accordance with the principles of the Declaration of Helsinki and was approved by the local ethics committee. Written informed consent was obtained from all patients prior to the procedure.

The study population consisted of consecutive patients admitted to the cardiology clinic with an indication for elective or urgent coronary angiography and who were deemed suitable for the transradial approach. Inclusion criteria were age ≥18 years, anatomical and clinical suitability for transradial access, and provision of informed consent for study participation. Exclusion criteria were defined as inadequate ulnar collateral circulation as assessed by the Allen or Barbeau test, previous radial artery intervention in the same extremity, upper extremity peripheral arterial disease or the presence of an arteriovenous fistula, cardiogenic shock or severe hemodynamic instability, a history of heparin-induced thrombocytopenia or known hypersensitivity to heparin, advanced renal failure (estimated glomerular filtration rate < 30 mL/min/1.73 m^2^), and refusal to participate in the study.

A total of 223 consecutive patients undergoing transradial coronary angiography were included in the analysis. Patients were assigned to two groups based on the route of heparin administration: the intravenous heparin group (IV group, *n* = 77) and the intra-arterial heparin group (IA group, *n* = 146). Group allocation was determined by operator preference; no randomization was performed.

All procedures were performed by two interventional cardiologists with extensive experience in transradial intervention. Radial artery puncture was carried out using the standard Seldinger technique after local anesthesia with 2–3 mL of 2% prilocaine. Following successful puncture, a 6-French hydrophilic-coated radial sheath was inserted in all patients. Immediately after sheath insertion, all patients received a standard IA spasmolytic cocktail consisting of 2.5 mg verapamil and 100 mcg nitroglycerin to prevent RAS.

The anticoagulation protocol was determined according to the heparin administration group. All patients initially received a standard dose of 5000 IU unfractionated heparin. In the IV group, heparin was administered as a bolus through a peripheral venous line, whereas in the IA group, the same dose was administered intra-arterially through the side port of the radial sheath. In patients requiring PCI, additional heparin doses were administered in accordance with current guideline recommendations to achieve target activated clotting time (ACT) levels.

For diagnostic coronary angiography, standard 6F-Judkins right and left catheters or, depending on operator preference, a Tiger catheter was used. In interventional procedures, guiding catheters appropriate to lesion anatomy were selected. The number of catheters used during the procedure, procedural duration (defined as the time from puncture to completion of the procedure), fluoroscopy time, total contrast volume, and total radiation dose were recorded prospectively.

The diagnosis of RAS was based on both clinical and operator-related criteria. Specifically, RAS was considered present when the patient reported newly developed forearm pain that increased during catheter manipulation, when the operator felt marked resistance during catheter advancement or withdrawal, and/or when severe spasm prevented completion of the procedure and required crossover to an alternative vascular access site, either femoral or contralateral radial. In cases where spasm was suspected, the radial artery was visualized to confirm the diagnosis and rule out other causes of resistance. All patients who underwent crossover due to severe spasm were classified as RAS positive. Importantly, vascular access crossover performed for reasons other than spasm (such as inability to successfully cannulate the coronary ostium or highly complex coronary anatomy) was not classified as RAS positive.

### Statistical Analysis

Statistical analyses were conducted using SPSS 28.0 software (IBM Corp., Armonk, NY, USA). The normality of the distribution for continuous variables was assessed using the Kolmogorov–Smirnov test. Normally distributed data were presented as mean ± standard deviation, and Student’s *t*-test was employed for inter-group comparisons. Non-normally distributed data were reported as median and interquartile range (25–75) and compared using the Mann–Whitney U test. Categorical variables were expressed as numbers and percentages and compared using the chi-square test. Receiver operating characteristics (ROC) curve was generated to define cut-off values (upper left corner of ROC as point of maximum sensitivity and specificity) of RAS in the study population. Univariate and multivariate logistic regression analyses were applied to identify independent predictors of RAS. Results with a *p*-value < 0.05 were considered statistically significant.

## 3. Results

**Figure 1:**
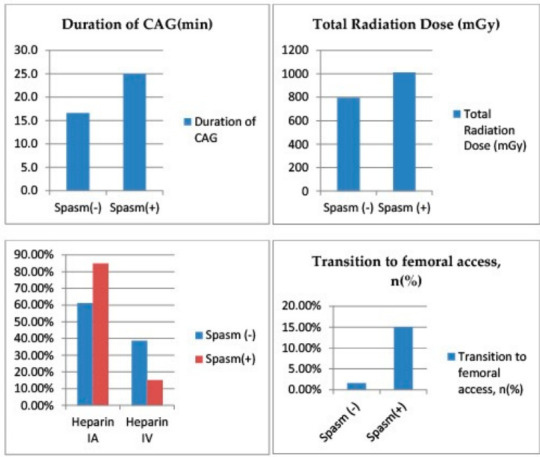
Statistically significant parameters between radial spasm-positive and spasm-negative groups. CAG: coronary angiography; IA: intra-arterial; IV: intravenous.

Baseline demographic, clinical, angiographic, and laboratory parameters are presented in Table 1. In the study population, heparin was administered intra-arterially (IA) to 146 patients and intravenously (IV) to 77 patients. In the study population, RAS was observed in 40 patients, while 183 patients did not experience RAS. There were no significant differences between the RAS positive and negative groups in terms of laboratory parameters. Patients with RAS were younger (*p* = 0.021). The number of catheters used was higher in these patients compared with those without spasm (*p* = 0.002). The number of patients who underwent PCI was higher and angiography duration was longer in the spasm-positive group (*p* = 0.025 and *p* < 0.001). The total radiation dose received by patients was higher in the spasm-positive group (*p* = 0.003). Total heparin dose administered was significantly higher in the spasm-positive group than in the spasm-negative group (8000 ± 2481 IU vs. 7186 ± 4009 IU, *p* = 0.019). The rate of crossover from the radial to the femoral access was higher in the radial spasm-positive group (*p* = 0.001). The rate of IA heparin administration was higher in the spasm-positive group, while the rate of IV heparin administration was higher in the spasm-negative group (*p* = 0.004) (Table 1, Figure 1).

**Figure 2:**
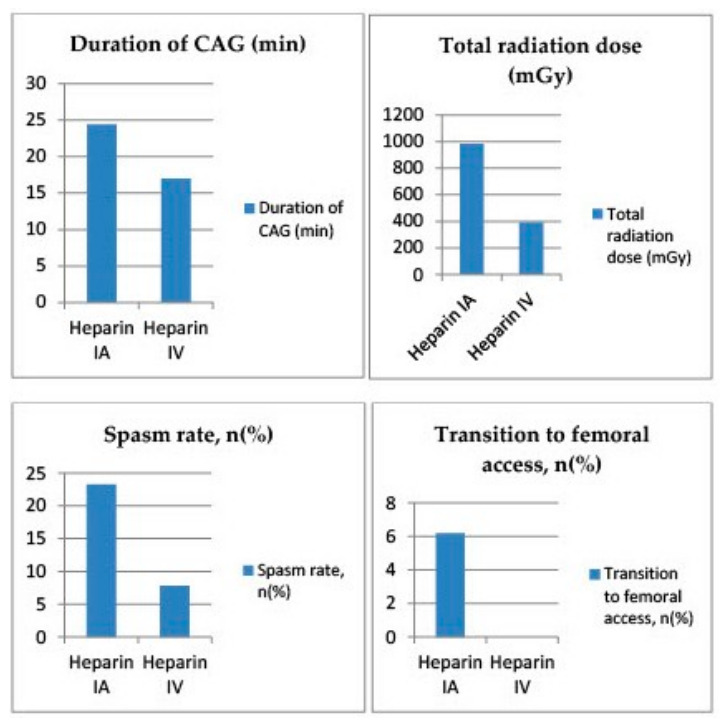
Statistically significant parameters between IA and IV heparin administration groups. CAG: coronary angiography; IA: intra-arterial; IV: intravenous.

There were no significant differences between the IA and IV heparin groups in terms of laboratory parameters. Body mass index (BMI) was higher in the IV heparin administration group, and family history rate was higher in these patients (*p* = 0.005 and *p* = 0.001). The group receiving IV heparin had a higher rate of patients presenting with STEMI (*p* = 0.011). There were no significant differences in other angiography indications. The duration of CAG was longer in the IA heparin group, and total radiation dose was higher in these patients (*p* = 0.003 and *p* = 0.019). RAS rate was higher in the IA heparin group (*p* = 0.004) and vascular access crossover was significantly higher in the radial spasm-positive group (*p* = 0.001). While all crossovers in the spasm-positive group (*n* = 6) were directly caused by severe RAS, the three crossovers observed in the spasm-negative group were strictly due to technical challenges, including the inability to engage the catheter into the coronary ostium and complex coronary anatomy (Table 2, Figure 2).

**Figure 3:**
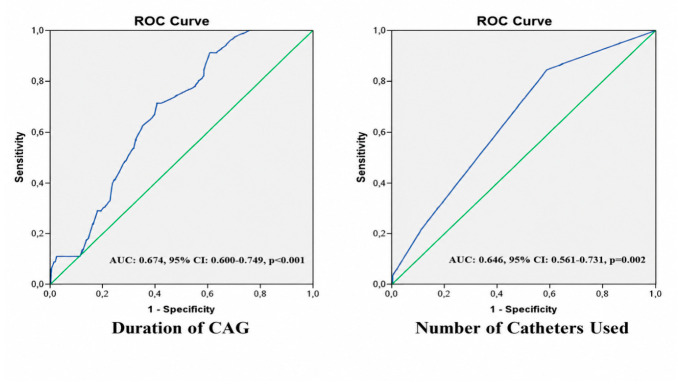
Receiver operating characteristics (ROC) curve analysis of duration of CAG and number of catheters used to identify radial artery spasm. The blue line represents the duration of CAG, and the red line represents the number of catheters used. AUC: area under curve; 95% CI: confidence interval; CAG: coronary angiography.

In the ROC curve analyses, AUC values for predicting RAS were significant for the number of catheters used and the duration of CAG (AUC: 0.674, 95% CI: 0.600–0.749, *p* < 0.001 and AUC: 0.646, 95% CI: 0.561–0.731, *p* = 0.002, respectively). The number of catheters used predicted RAS with a sensitivity of 84.4% and specificity of 59.0%. The cut-off level of >13.5 min duration of CAG predicted RAS with a sensitivity of 82.2% and specificity of 58.5% (Figure 3).

Univariate and multivariate binary logistic regression analysis were performed to investigate independent correlates of RAS in the study population (Table 3). In the univariate model, age, duration of angiography, number of catheters used, IA heparin administration, and PCI were found to have a significance in differentiating between patients who developed and did not develop RAS. In the multivariate model, the number of catheters used and IA heparin administration were found to be independent predictors of RAS (Table 3).

## 4. Discussion

The results of our study showed that RAS was associated with age, number of catheters used, angiography duration, total radiation dose, and IA heparin administration. Additionally, these parameters had an acceptable correlation with RAS. IA heparin administration, number of catheters used, and the duration of CAG had acceptable values of sensitivity and specificity in predicting RAS. In multivariate regression analysis, number of catheters used and IA heparin administration were potential independent predictors of RAS.

In transradial interventions, heparin is primarily used to prevent radial artery occlusion (RAO) and thromboembolic complications. Several studies have compared IA and IV heparin with respect to RAO and have generally shown similar efficacy between the two routes [15,16,17]. However, most of these studies did not consider RAS as a primary result and instead evaluated it as a secondary or incidental parameter. In this regard, our study contributes to the literature by directly examining the effect of the heparin administration route on RAS.

The mechanisms underlying the higher incidence of RAS associated with IA heparin administration may be explained by the physiological and structural properties of the radial artery. The radial artery is highly sensitive to vasoconstrictive stimuli because of its thick tunica media and high density of alpha-1 adrenergic receptors [9,18]. Intra-arterially administered heparin comes into direct contact with the vessel wall and may cause local endothelial irritation and a reduction in nitric oxide (NO) bioavailability. A decrease in endothelium-derived NO release may enhance the contractile response of vascular smooth muscle cells and facilitate vasospasm [19]. In contrast, when administered intravenously, heparin enters the systemic circulation and reaches the radial artery in a more diluted form, thereby minimizing local irritative effects. This mechanism may explain why IV heparin was associated with lower spasm rates in our study.

Our finding that the number of catheters used independently predicted RAS further highlights the importance of mechanical factors. Repeated catheter exchanges may result in recurrent friction and micro trauma to the vessel wall, thereby promoting endothelial dysfunction and increasing the likelihood of spasm. This observation is consistent with the findings reported by Ho et al. [20] and Sandoval et al. [21]. Therefore, in patients with complex coronary anatomy, minimizing catheter exchanges and using single-catheter strategies whenever feasible may be important for reducing the risk of RAS [22].

We also observed significant associations between younger age, longer procedural duration, higher radiation dose, and the occurrence of RAS. Younger patients may be more prone to spasm because of greater vascular reactivity and more pronounced sympathetic activity. However, the fact that these variables did not remain independent predictors in the multivariable analysis suggests that their influence may be mediated indirectly through procedural factors. The longer procedure duration and higher radiation exposure observed in the IA heparin group are likely attributable to the higher incidence of RAS in this group, as spasm prolongs catheter manipulation and may necessitate additional procedural steps. Similarly, the significantly higher total heparin dose observed in the spasm-positive group is likely a consequence of prolonged catheter manipulation and the higher rate of percutaneous coronary interventions performed in these patients, which routinely necessitate supplemental anticoagulation in daily practice. From a clinical perspective, RAS is not merely a source of patient discomfort, it is also a relevant procedural complication that prolongs intervention time, increases radiation exposure, and compromises procedural success. In severe cases, it may necessitate crossover to femoral access, thereby negating the major advantages of the transradial approach. Preventing RAS is therefore critical to the overall success of transradial procedures.

Our findings suggest that the IV administration of heparin may reduce the risk of RAS without requiring any additional cost or technical complexity. This implies that a simple modification of routine procedural protocol may result in clinically meaningful benefits. In patients considered to be at particularly high risk of spasm, such as younger individuals, those with smaller radial artery diameter, or anxious patients, preferential use of IV heparin may provide even greater benefit.

Several limitations of our study should be acknowledged. First, because this was a single-center study, the generalizability of the findings may be limited. Second, the diagnosis of RAS was based on clinical and operator-related criteria and was not confirmed using objective imaging modalities. Third, only a fixed heparin dose was used, and the effects of alternative dosing strategies were not assessed. Fourth, due to the observational design of our study, causality cannot be firmly established. Variables such as procedure duration, radiation dose, and the number of catheters used may be both causes and consequences of RAS. Therefore, our findings should be interpreted as associations rather than causal relationships. Fifth, baseline differences were observed between the IA and IV heparin groups, including BMI, family history, and STEMI presentation. Although our multivariate analysis adjusted for several covariates, residual confounding cannot be entirely excluded. A randomized controlled trial would be required to eliminate these imbalances. No a priori sample size calculation was performed, as this was an observational study of consecutive patients. A post-hoc power analysis was not considered necessary for the purpose of this exploratory analysis. Nevertheless, the prospective design represents a major strength of the study.

## 5. Conclusions

In conclusion, our study demonstrates an association between IV heparin administration and a significantly lower incidence of RAS, as well as a reduced need for femoral crossover, when compared with IA administration in patients undergoing transradial coronary angiography. IA heparin administration and the number of catheters used during the procedure were identified as potential independent predictors of RAS. However, due to the observational design, causality cannot be firmly established, and these findings should be interpreted as associations. As one of the first studies in the literature to evaluate the direct effect of the heparin administration route on RAS as a primary endpoint, our findings emphasize that IV heparin may reduce the risk of spasm without adding cost or procedural difficulty. This simple modification in procedural protocol may offer an effective and easily applicable strategy to improve procedural success and patient comfort, particularly in patients at high risk for RAS.

## Figures and Tables

**Table 1:** Baseline clinical, catheterization, and laboratory characteristics of patients who developed and did not develop radial artery spasm.

Variables		Radial Spasm (−) Group (*n* = 183)	Radial Spasm (+) Group (*n* = 40)	*p*
Age (years)		58.6 ± 11.2	55.0 ± 10.6	0.021
Gender, male, *n* (%)		113 (61.7)	18 (45.0)	0.051
BMI (kg/m^2^)		28.8 ± 4.2	28.6 ± 4.1	0.950
Hypertension, *n* (%)		116 (63.4)	23 (57.5)	0.486
Diabetes mellitus, *n* (%)		82 (44.8)	12 (30.0)	0.086
Hyperlipidemia, *n* (%)		76 (41.5)	14 (35.0)	0.446
Smoking, *n* (%)		73 (39.9)	14 (35.0)	0.566
Family history, *n* (%)		68 (37.2)	13 (32.5)	0.579
CAD history, *n* (%)		71 (38.8)	12 (30.0)	0.297
Hgb (g/dL)		13.8 ± 1.8	13.5 ± 1.6	0.308
Platelets (10^3^/µL)		271.8 ± 83.8	279.8 ± 75.0	0.342
Glucose (mg/dL)		148.4 ± 73.1	149.8 ± 83.1	0.526
Urea (mg/dL)		33.1 ± 12.1	34.3 ± 13.3	0.378
Creatinine (mg/dL)		0.91 ± 0.25	0.92 ± 0.30	0.824
Albumin (g/L)		42.0 ± 3.5	42.1 ± 4.2	0.917
TG (mg/dL)		175.0 (142.8–214.8)	190.0 (169.7–228.4)	0.761
LDL (mg/dL)		109.4 ± 39.5	113.1 ± 47.7	0.795
HDL (mg/dL)		42.9 ± 10.4	41.0 ± 9.9	0.361
CRP (mg/L)		8.3 (3.0–16.5)	4.8 (3.5–7.4)	0.499
Troponin I (ng/L)		80.5 (25.0–162.0)	33.1 (10.8–61.8)	0.137
Indication of CAG				
-Elective patient-diagnostic CAG, *n* (%)		132 (72.1)	32 (80.0)	0.372
-STEMI, *n* (%)		5 (2.7)	1 (2.5)	0.932
-Non-STEMI, *n* (%)		25 (13.7)	3 (7.5)	0.287
-USAP, *n* (%)		21 (11.5)	4 (10.0)	0.789
Barbeau test, *n* (%)	Type A Type B	165 (90.2) 18 (9.8)	32 (80.0) 8 (20.0)	0.070
Catheter width (F), *n* (%)	5F 6F	14 (7.7) 169 (92.3)	1 (2.5) 39 (97.5)	0.239
Number of catheters used		1.69 ± 0.69	2.05 ± 0.68	0.002
Duration of CAG (min)		16.6 (14.0–20.3)	25.0 (20.5–29.3)	<0.001
Total radiation dose (mGy)		796.0 (436.0–1494.0)	1011.0 (659.0–1222.0)	0.003
PCI (%)		70 (38.3)	23 (57.5)	0.025
Total heparin dose (IU)		7186 ± 4009	8000 ± 2481	0.019
Heparin administration, *n* (%)	IA IV	112 (61.2) 71 (38.8)	34 (85.0) 6 (15.0)	0.004
All-cause crossover to femoral access, *n* (%)		3 (1.6)	6 (15.0)	0.001

**Abbreviations:** BMI: body mass index; CAD: coronary artery disease; Hgb: hemoglobin; TG: triglyceride; LDL: low density lipoprotein; HDL: high density lipoprotein; CRP: C-reactive protein; CAG: coronary angiography; STEMI: ST-elevated myocardial infarction; Non-STEMI: myocardial infarction without ST elevation; USAP: unstable angina pectoris; F: French; PCI: percutaneous coronary intervention; IA: intra-arterial; IV: intravenous. Values are presented as mean ± standard deviation, number (percentage), and median (quartile).

**Table 2:** Baseline clinical, catheterization, and laboratory characteristics of IA and IV heparin administration groups.

Variables		IA Heparin Administration Group (*n* = 146)	IV Heparin Administration Group (*n* = 77)	*p*
Age (years)		57.7 ± 11.2	58.4 ± 11.0	0.706
Gender, male, *n* (%)		92 (63.0)	39 (50.6)	0.075
BMI (kg/m^2^)		28.3 ± 4.2	29.7 ± 4.0	**0.005**
Hypertension, *n* (%)		93 (63.7)	46 (59.7)	0.562
Diabetes mellitus, *n* (%)		59 (40.4)	35 (45.5)	0.468
Hyperlipidemia, *n* (%)		63 (43.2)	27 (35.1)	0.242
Smoking, *n* (%)		62 (42.5)	25 (32.5)	0.146
Family history, *n* (%)		38 (26.0)	43 (55.8)	**0.001**
CAD history, *n* (%)		48 (32.9)	35 (45.5)	0.065
Hgb (g/dL)		13.9 ± 1.8	13.6 ± 1.6	0.148
Platelets (10^3^/µL)		274.0 ± 84.1	271.6 ± 78.8	0.768
Glucose (mg/dL)		149.2 ± 78.1	147.5 ± 68.0	0.621
Urea (mg/dL)		33.8 ± 12.5	32.3 ± 11.8	0.355
Creatinine (mg/dL)		0.93 ± 0.25	0.88 ± 0.28	0.063
Albumin (g/L)		42.1 ± 3.9	41.8 ± 3.1	0.455
TG (mg/dL)		175.0 (152.0–217.4)	180.0 (140.3–216.8)	0.767
LDL (mg/dL)		111.5 ± 40.1	107.1 ± 42.8	0.376
HDL (mg/dL)		42.5 ± 9.9	42.6 ± 11.2	0.982
CRP (mg/L)		9.0 (3.0–18.2)	5.1 (3.3–6.1)	0.523
Troponin I (ng/L)		72.8 (28.1–155.4)	72.4 (29.8–144.6)	0.114
Indication of CAG				
-Elective patient-diagnostic CAG, *n* (%)		110 (75.3)	54 (70.1)	0.184
-STEMI, *n* (%)		1 (0.7)	5 (6.5)	**0.011**
-Non-STEMI, *n* (%)		20 (13.7)	8 (10.4)	0.478
-USAP, *n* (%)		15 (10.3)	10 (13.0)	0.541
Barbeau test, *n* (%)	Type A Type B	131 (89.7) 15 (10.3)	66 (85.7) 11 (14.3)	0.375
Catheter width (F), *n*(%)	5F 6F	12 (8.2) 134 (91.8)	3 (3.9) 74 (96.1)	0.220
Number of catheters used		1.76 ± 0.71	1.74 ± 0.70	0.900
Duration of CAG (min)		24.4 ± 19.7	17.0 ± 11.4	**0.003**
Total radiation dose (mGy)		984.4 (520.5–1724.8)	390.7 (286.5–531.5)	**0.019**
PCI (%)		63 (43.2)	30 (39.0)	0.546
Total heparin dose (IU)		7226 ± 2493	7532 ± 3479	0.599
Spasm rate, *n* (%)		34 (23.3)	6 (7.8)	**0.004**
Crossover to femoral access, *n* (%)		9 (6.2)	0 (0.0)	**0.026**

**Abbreviations:** IA: intra-arterial; IV: intravenous; BMI: body mass index; HT: hypertension; DM: diabetes mellitus; HPL: hyperlipidemia; CAD: coronary artery disease; Hgb: hemoglobin; TG: triglyceride; LDL: low density lipoprotein; HDL: high density lipoprotein; CRP: C-reactive protein; CAG: coronary angiography; STEMI: ST-elevated myocardial infarction; Non-STEMI: myocardial infarction without ST elevation; USAP: unstable angina pectoris; F: French; PCI: percutaneous coronary intervention. Values are presented as mean ± standard deviation, number (percentage), and median (quartile). Bold fonts indicate a *p* value lesser than 0.05.

**Table 3:** The predictors of radial artery spasm in binary logistic regression analysis.

Variables	Unadjusted OR (95% CI)	*p*	Adjusted OR (95% CI)	*p*
Age	0.972 (0.903–1.002)	**0.048**		
The duration of angiography	1.025 (1.007–1.042)	**0.005**		
Number of catheters used	2.021 (1.257–3.267)	**0.004**	2.072 (1.252–3.429)	**0.005**
IA heparin administration	0.278 (0.111–0.697)	**0.006**	0.225 (0.083–0.610)	**0.003**
PCI	2.184 (1.091–4.373)	**0.027**		
Total heparin dose	1.000 (1.000–1.000)	0.259		

**Abbreviations:** OR: odds ratio; CI: confidence intervals; IA: intra-arterial; PCI: percutaneous coronary intervention. Bold fonts indicate a *p* value lesser than 0.05.

## Data Availability

The datasets used and/or analyzed during the current study are available from the corresponding author on reasonable request.

## References

[1] Campeau L. (1989). Percutaneous radial artery approach for coronary angiography. Catheter. Cardiovasc. Diagn..

[2] Kiemeneij F., Laarman G.J. (1993). Percutaneous transradial artery approach for coronary stent implantation. Catheter. Cardiovasc. Diagn..

[3] Ferrante G., Rao S.V., Jüni P., Da Costa B.R., Reimers B., Condorelli G., Anzuini A., Jolly S.S., Bertrand O.F., Krucoff M.W. (2016). Radial versus Femoral Access for Coronary Interventions Across the Entire Spectrum of Patients With Coronary Artery Disease: A Meta-Analysis of Randomized Trials. JACC. Cardiovasc. Interv..

[4] Jolly S.S., Yusuf S., Cairns J., Niemelä K., Xavier D., Widimsky P., Budaj A., Niemelä M., Valentin V., Lewis B.S. (2011). Radial versus femoral access for coronary angiography and intervention in patients with acute coronary syndromes (RIVAL): A randomised, parallel group, multicentre trial. Lancet.

[5] Valgimigli M., Gagnor A., Calabró P., Frigoli E., Leonardi S., Zaro T., Rubartelli P., Briguori C., Andò G., Repetto A. (2015). Radial versus femoral access in patients with acute coronary syndromes undergoing invasive management: A randomised multicentre trial. Lancet.

[6] Ibanez B., James S., Agewall S., Antunes M.J., Bucciarelli-Ducci C., Bueno H., Caforio A.L.P., Crea F., Goudevenos J.A., Halvorsen S. (2018). 2017 ESC Guidelines for the management of acute myocardial infarction in patients presenting with ST-segment elevation: The Task Force for the management of acute myocardial infarction in patients presenting with ST-segment elevation of the European Society of Cardiology (ESC). Eur. Heart J..

[7] Byrne R.A., Rossello X., Coughlan J.J., Barbato E., Berry C., Chieffo A., Claeys M.J., Dan G.A., Dweck M.R., Galbraith M. (2023). 2023 ESC Guidelines for the management of acute coronary syndromes. Eur. Heart J..

[8] Mason P.J., Shah B., Tamis-Holland J.E., Bittl J.A., Cohen M.G., Safirstein J., Drachman D.E., Valle J.A., Rhodes D., Gilchrist I.C. (2018). An Update on Radial Artery Access and Best Practices for Transradial Coronary Angiography and Intervention in Acute Coronary Syndrome: A Scientific Statement from the American Heart Association. Circ. Cardiovasc. Interv..

[9] Zus A.S., Crișan S., Luca S., Nișulescu D., Valcovici M., Pătru O., Lazăr M.-A., Văcărescu C., Gaiță D., Luca C.-T. (2024). Radial artery spasm. A review on incidence, prevention and treatment. Diagnostics.

[10] Jan R., Andrzej T., Artur D., Szymon G., Artur P., Karol S., Januszek R., Rzeszutko Ł., Surdacki A., Bartuś S. (2024). Radial artery spasms angiographic morphology, risk factors and management. Postep. Kardiol. Interwencyjnej.

[11] Curtis E., Fernandez R., Khoo J., Weaver J., Lee A., Halcomb E. (2023). Clinical predictors and management for radial artery spasm: An Australian cross-sectional study. BMC Cardiovasc. Disord..

[12] Riangwiwat T., Blankenship J.C. (2021). Vascular complications of transradial access for cardiac catheterization. US Cardiol. Rev..

[13] Kiemeneij F., Vajifdar B.U., Eccleshall S.C., Laarman G.J., Slagboom T., van der Wieken R. (2003). Evaluation of a spasmolytic cocktail to prevent radial artery spasm during coronary procedures. Catheter. Cardiovasc. Interv..

[14] Kwok C.S., Rashid M., Fraser D., Nolan J., Mamas M. (2015). Intra-arterial vasodilators to prevent radial artery spasm: A systematic review and pooled analysis of clinical studies. Cardiovasc. Revasculariz. Med..

[15] Pancholy S.B. (2009). Comparison of the effect of intra-arterial versus intravenous heparin on radial artery occlusion after transradial catheterization. Am. J. Cardiol..

[16] Almansori M., Ouf S. (2014). Comparison between intravenous versus intra-arterial heparin during transradial coronary artery catheterization. J. Saudi Heart Assoc..

[17] Khalid W., Saif M., Halim A., Janjua A.F., Khan K.A., Rauf A., Aziz Z., Aziz S. (2020). Comparison of intravenous versus intra-arterial heparin for the prevention of radial artery occlusion during transradial coronary artery catheterization. Pak. Armed Forces Med. J..

[18] Coghill E.M., Johnson T., Morris R.E., Megson I.L., Leslie S.J. (2020). Radial artery access site complications during cardiac procedures, clinical implications and potential solutions: The role of nitric oxide. World J. Cardiol..

[19] Hubert A., Seitz A., Pereyra V.M., Bekeredjian R., Sechtem U., Ong P. (2020). Coronary Artery Spasm: The Interplay between Endothelial Dysfunction and Vascular Smooth Muscle Cell Hyperreactivity. Eur. Cardiol..

[20] Ho H.H., Jafary F.H., Ong P.J. (2012). Radial artery spasm during transradial cardiac catheterization and percutaneous coronary intervention: Incidence, predisposing factors, prevention, and management. Cardiovasc. Revasculariz. Med..

[21] Sandoval Y., Bell M.R., Gulati R. (2019). Transradial Artery Access Complications. Circ. Cardiovasc. Interv..

[22] Lin Y.C., Chen Y.C., Tu C.M., Chang Y.Y., Hsu J.C., Wu Y.W., Li A.H. (2026). Differential Predictors of Severe Versus Milder Radial Artery Spasm: Insights from a Large Contemporary Distal Radial Access Cohort. Catheter. Cardiovasc. Interv. Off. J. Soc. Card. Angiogr. Interv..

